# The Hospital. Nursing Section

**Published:** 1905-05-06

**Authors:** 


### The Hospital.

### Nursing Section.

Contributions for this Section of "The Hospital" should be addressed to the Editor, "The Hospital" Nursing Section, 28 & 29 Southampton Street, Strand, London, W.C.

No. 971.—Vol. XXXVIII. SATURDAY, MAY 6, 1905.

### Notes on News from the Nursing World.

### QUEEN VICTORIA'S JUBILEE INSTITUTE FOR NURSES.

The report of Queen Victoria's Jubilee Institute for Nurses for 1904, which has just been issued, must be regarded from two points of view. As to the appreciation of the work of the Institute in the country, there is no room for doubt. A remarkable proof of this is supplied in the report. A town entirely composed of working men and their families, employed chiefly in large railway works, has just obtained the services of a Queen's nurse. It took the committee two years to raise the necessary funds in their off-duty hours. The figures themselves show that the number of Queen's nurses on the roll at the end of the year was 1,121 as against 1,024 in 1903; that 41 Queen's nurses rejoined the Institute during 1904, and that 122 nurses received certificates on the satisfactory completion of the required term of service. There are now no fewer than 660 distinct associations affiliated to the Institute, and 14 county nursing associations employing 254 village nurses, in addition to 40 Queen's nurses. Clearly, the movement meets a great public want, and its growth is likely to continue, always supposing that it is adequately provided with the sinews of war. Here, we regret to say, the report is not satisfactory, and the Council confess that their chief anxiety at the present time is the financial position of the Institute. At the end of 1905 the sum collected in commemoration of the Diamond Jubilee, which has been drawn upon year by year, will be exhausted, and a serious deficit will thus have very shortly to be faced. In view of this contingency the Council have reluctantly, though wisely, already resolved upon certain economies, but even the reduction of the payments for district training and the discontinuance of training in hospitals, leaves the estimated requisite expenditure of 1905 £2,000 in excess of receipts. We are glad to learn that a special effort is to be put forward to obtain increased funds, and we hope that those who, while regarding the Jubilee Institute as a most fitting memorial of Queen Victoria, have either not subscribed to it as yet, or subscribed a very meagre sum, will lose no time in doing their best to relieve the anxiety of the Council, either by sending in their names as contributors, or by doubling their subscriptions.

### LADY DUDLEY'S FUND.

Accompanying the report of Queen Victoria's Jubilee Institute, is a statement of the accounts of Lady Dudley's Fund for the establishment of district nurses in the poorest part of Ireland covering a period of 21 months. The cost of the existing staff of ten nurses is between £1,200 and £1,300 a year. The amount contributed was £918 in subscriptions and £9,213 in donations. The Fund has therefore a considerable capital. But it is clear that unless the ordinary receipts suffice to defray the ordinary expenses, there will be a frequent necessity for the sale of investments, which as far as possible should be avoided.

### UNCERTIFICATED NURSES.

Last week a correspondent commented on a newspaper report of the evidence of the matron of the London Hospital before the Select Committee of the House of Commons on Registration, in which the remark was attributed to her, "I get as many requests for nurses who are uncertificated as for those who are certificated." Our correspondent, in expressing surprise at the statement, omitted to add that Miss Lückes also said that, "she thought that all nurses should have a certificate of some sort." She was not therefore committing herself, even by inference, to the advocacy of the employment of uncertificated nurses. On this point, whatever may be the feeling about registration, there is no room for difference of opinion. No nurse who cannot produce her certificate of training ought to be employed on a case under any circumstances whatever, and if the production of it were always required by the friends of a patient as well as insisted upon by the medical man in attendance, we believe that the number of half-trained persons would soon be diminished. The existence of the uncertificatd nurse is a menace to the safety of the community.

### AN EXCESS OF DISCIPLINE.

Under the heading of "Silence at Meals," a Poor-law Infirmary nurse writes us to-day, stating that the assistant matron of the institution to which she is attached insists upon the nurses observing silence at meals. Our correspondent, therefore, inquires whether it is a breach of hospital etiquette to talk at meals, and whether the assistant matron has the power to enforce the rule prohibiting it. There are, of course, obvious reasons why there should be no noisy conversation at the breakfast or dinner table, but our correspondent especially guards herself against the advocacy of want of restraint, and in our opinion a quiet interchange of remarks is not only not a violation of hospital etiquette but is absolutely to be desired. It should, we think, be recognised that nurses who are on duty for nearly twelve hours without a break —a state of affairs which ought to be altered—are entitled to a little relaxation when they meet at meals, and to impose silence upon them savoursof tyranny, while it is also bad for their digestion, inasmuch as it tends to cause undue haste in the consumption of food. An assistant matron has usually no power to make rules, and we presume that in the case mentioned by our correspondent, she is acting under instructions. If not, an appeal to her official superior should be made, and in the last resort the guardians might be asked to intervene.

### THE ABUSE OF NURSING ASSOCIATIONS.

An important point was raised at the annual meeting of the Carlisle District Nursing Association the other day. The honorary physician, in seconding the adoption of the report, said he had a hint that sometimes the people who availed themselves of the benefits of the Association were not the persons for whom it was intended—the sick poor. In fact, he had heard that it was suffering through being utilised by individuals who could afford to pay for nursing. Another side of the question was dealt with by a subsequent speaker, who urged that outside the absolutely poor is a large class who require help as much as the working man, and he also contended that the payment of £24 by patients voluntarily proved that recognition was made of services rendered. We agree with the view that the poor young shopman, or girl in a shop, or people who have to keep up a respectable appearance, are often unable to pay for nursing, and ought, if possible, to be nursed gratuitously. But we are afraid that the committee of the Carlisle Association are not singular in suffering from the abuse of a nursing charity. This is, in fact, one of the dangers which has to be guarded against, and it cannot be too strongly insisted that when a district nurse is attending a case—inquiries cannot always be made before she goes—an official of the Association ought to able to ascertain whether the friends of the patient are in a position to pay, and, in that event, to remind them of their obligation, unless it is fulfilled spontaneously.

### NURSING CHINESE NATIVES.

It will be observed that our contributor who describes to-day a visit to a Chinese native hospital mentions that except in the female wards all the attendants are men, and that in the latter native women act as nurses. As English sisters are employed in the Government Civil Hospital at Hongkong, and attend there equally upon native as well as upon foreign patients it may be assumed that there is no rooted antipathy to their ministrations. That the nurses in the native-managed hospitals ought to be efficiently trained would probably be admitted by the Tung Wah Hospital committee, and we hope that in process of time the advisability of substituting them for untrained attendants will be formally recognised and acted upon.

### QUEEN ALEXANDRA'S MILITARY NURSING SERVICE.

The following staff nurses attached to Queen Alexandra's Imperial Military Nursing Service have been promoted to be sisters:—Misses A. R. F. Auchmuty, S. K. Bills, B. N. Daker, G. Knowles, C. Mackay, W. G. Massey, B. Rennie, and M. Worthington. The period of provisional service as staff nurse of Misses G. M. Allen, K. M. Bulman, E. Foster, M. E. M. Grierson, and A. M Orchard having expired, they have been confirmed in their appointments. Miss Sophia Orpen Beamish has been appointed provisionally staff nurse.

### ROYALTY AND DISTRICT NURSING.

Princess Louise has promised to be present at a fête to be held in the grounds of Carnforth Lodge, Hammersmith, on Monday, June 19th, in aid of the Hammersmith and District Nursing Association. The Bishop of London and the members for Hammersmith and Fulham will be present to receive her Royal Highness. A number of people have already undertaken to collect purses of money, which will be presented by children to the Princess, each purse to contain not less than three guineas. It is hoped that a substantial sum will be realised for the benefit of the organisation, which is badly in need of financial help.

### NURSING IN FIJI.

We are informed that the Secretary of State for the Colonies has approved the appointment of Miss May Christina Anderson, R.R.C., matron of the Colonial Hospital at Suva, Fiji, and Miss Maud Lilian Anderson, senior staff nurse of the Colonial Hospital, to be respectively matron and staff nurse of the Public Lunatic Asylum, Fiji. The work is to be undertaken in conjunction with their other duties, but the Colonial Secretary has also approved the appointment of Miss Florence Evelyn Holmes to be junior staff nurse at the Hospital.

### NEW CONVALESCENT HOME AT LEICESTER.

There was opened on Monday, for the benefit of working men of Leicester, a very handsome and completely equipped Convalescent Home, at a cost of £12,500. Desford Hall, with the grounds surrounding it, has been purchased and converted into an institution capable of accommodating 50 patients, whose limit of stay will be three weeks, unless a medical man advises that a second period would restore him to health. The Home having been established by the aid of money raised from all sections of the community through the Queen Victoria Memorial Fund, is to be maintained by an organised system of weekly contributions from the workers themselves. It is in charge of a fully-trained nurse, the first matron being Miss Havens, who has been at her post for some days. Miss Havens is well known and exceedingly popular in Leicester, for she was Sister Lucy of the Accident Ward at Leicester Infirmary for eight years, and working men who have already been patients under her care are delighted that the management of the new Home has been entrusted to her.

### PRIZE DISTRIBUTION AT SALOP INFIRMARY.

During the past year a code of instruction has been provided for the nursing staff of the Salop Infirmary. Lectures and classes have been given in anatomy and physiology, surgery, gynæcology, gynæcological nursing, bandaging, and practical nursing. Examinations have been held upon these subjects, and the results have been satisfactory. The system of practical and theoretical instruction will, for the future, be maintained each year. The authorities view with satisfaction the work accomplished by the members of the nursing staff. It is also gratifying to know that the nurses greatly appreciate the further advantages which are added to their general training. The prize distribution took place on Saturday afternoon, when a number of friends and supporters of the infirmary met together in the board-room, by invitation of the matron, Miss F. G. Pegg. An interesting programme consisting of vocal and instrumental music was provided. Speeches were made by Mr. Nicholas Robinson, chairman of the board, and by the chaplain, Archdeacon Maude. Mrs. Robinson distributed the prizes. The successful nurses who gained the prizes were Nurses Foley and Fulford, equal, first prize, physiology and anatomy; Nurse Vaughan, first, surgery, Nurse Griffiths, second; Nurse White, first, gynæcology, Nurse Camm second; Nurse Vaughan, first, gynæcological nursing, Nurse Griffiths, second; Nurse Dickson, first, bandaging (seniors), Nurse Wallis, second; Nurses Newsham and Robbins, first, bandaging (juniors), Nurses Bennett and Tuckler, second; Nurse Vaughan, first, practical nursing, Nurse Dickson, second. One of the recreations of the nurses during the summer months is gardening, and the matron gives a prize to the nurse who has the best and tidiest garden during the year, marks being awarded each week after inspection. The prize was won this year by Nurse Every.

### MATERNITY EXTENSION AT CARDIFF.

The new maternity department of the Cardiff branch of the Queen's Institute in St. Andrew's Crescent has just been opened by the Mayoress. The importance of the new department is emphasised in the annual report of the association. For the present the staff consists of the maternity superintendent and one midwife, but it will be necessarily augmented at a very early date. During the year the nurses were increased from six to eight, and there are also four others with full hospital training working under the auspices of the committee. An appeal has been issued for further financial assistance, and it is pointed out that in addition to the extra expenditure of £150 last year the work is still growing.

### LEGACY FOR A NURSE.

The late Mr. Joseph Procter Mann, of The Cottage, Moreton-in-the-Marsh, Gloucestershire, whose estate was valued at upwards of £43,000, bequeathed a thousand pounds to Miss Lizzie Bayliss, who, it is stated in the will, nursed him through a long and serious illness. This is an unusually handsome recognition of services, and shows that they must have been as ungrudgingly rendered as they were warmly appreciated.

### DISTRICT NURSES FOR EMERGENCY WORK.

At the first annual meeting of the Devonshire Nursing Association, over which Lord Clifford presided, a resolution recommending union with the Somerset and Cornwall Nursing Associations was passed. It is proposed that there should be founded, at the joint expense of the three Associations, a home or homes, at some centre or centres convenient to the three counties, for the purpose of establishing a staff of nurses available for emergency work. These nurses, when not required for such work in connection with affiliated districts, are to be open for employment in approved unaffiliated districts. The committee were also empowered at the meeting to take into consideration the establishment of a joint training home in connection with one or all of the three counties. The idea is that the Associations can train their own nurses for much less money than they can be trained elsewhere. This may be so; but we hope that efficiency will be studied even before economy. District nurses undertaking emergency work should, as far as possible, be fully trained.

### A NURSE'S ALBUM DURING THE BOER WAR.

Among the members of the Army Nursing Service Reserve who served in the Transvaal during the whole of the Boer war was Sister A. K. Statham. She was attached to No. 1 General Hospital, first at Naauport and then, when it moved on, at Johannesburg. For the admirable work she did between 1899 and 1902 Miss Statham was awarded two war medals, and she has another souvenir of that exciting period in her experiences in the shape of an album which she kept for her patients, if they felt so disposed, to write something in. Upwards of a hundred contributed, and the album was found so interesting that it has lately been printed and circulated amongst friends of the owner. We have seen a copy, and the contents certainly form an extremely interesting record in prose and verse of many incidents of the war.

### EXAMINATION AT FULHAM INFIRMARY.

Certificates have just been presented to six nurses trained in the Fulham Poor-law Infirmary who passed an examination. The names of the successful probationers are as follows:—B. Healey, S. Hughes, B. A. Stidstone, M. E. Dampney, E. Pedley, and E. E. Blackmore.

### BOYS IN PETTICOATS.

A district nurse's experiences are sometimes very amusing. In Connemara, for instance, in some of the districts a nurse has met with boys of 12 and 14 in petticoats. The mothers insist that the petticoats are worn to prevent the fairies from taking their boys, but the common-sense nurse often attributes the custom to motives of economy.

### A NEW ASSOCIATION IN LINCOLNSHIRE.

It has been decided to extend the work of the Lincolnshire Nursing Association in North Lincolnshire, and to group the villages of Caenby, Glentham, Saxby, Normanby, Owmby, and other parishes together in a rural district in order that a nurse may be located in their midst. At a meeting held for the purpose of initiating the movement, Miss Glover, superintendent nurse of the Lincolnshire Association, said that if £65 a year were raised it would cover the cost to the district of the nurse, her bicycle, and the outfit of appliances necessary, and it is believed that there will be no serious difficulty in raising this sum for the purpose.

### SHORT ITEMS.

Miss E. A. Lake, who was trained at the Royal Hants County Hospital, Winchester, afterwards working on their private staff for 14 years, has resigned, and joined Miss Mocatta at Grosvenor House, Southampton.

### The Nursing Outlook.

"From magnanimity, all fear above;
From nobler recompense, above applause,
Which owes to man's short outlook all its charm."

### HOSPITAL-TRAINED NURSES AND PRIVATE CASES.

We propose to consider why hospital-trained nurses sometimes fail in private cases. The primary reason for such failure is undoubtedly due to the fact that many nurses are not taught to consider the patients sufficiently as individuals. In a busy hospital, full of patients, the latter are apt to be regarded too much as cases, and the interest they excite may be due in large measure to the ailment from which they are suffering. In private houses, where the nurse has charge of one case only, it is essential to success that she shall study the individual, and make her patient feel that her main object is to co-operate with the doctor in promoting speedy recovery, whilst her immediate and constant purpose is to make her patient as comfortable always as circumstances will permit. No woman ought to undertake the duties of a private nurse unless she is endowed with considerable sympathy, and feels that her mission is to minister, to the happiness of each individual patient, who may be placed in her charge. A failure to appreciate these simple but necessary requirements may be held to largely account for the shortcomings of the majority of nurses who become unpopular in private work.

The initial fault must be put down to the training school, especially where a school for medical students is attached, where much time is devoted to the preparation and clearing away for clinical classes, where the nurses have few opportunities of dressing or really attending to the cases in the sense they have to attend to them in private houses. In such large establishments the tendency too often is to make ward work and ward smartness the first considerations, rather than the actual nursing. On the other hand, in those hospitals where there is no medical school attached, though the nurses may not see such varied treatment, they do considerably more nursing in every sense of the word. Another cause is due to the altered circumstances of treatment in busy hospitals, where under the new system convalescent and chronic cases cannot be retained. In such establishments few if any of the probationers see or are taught the care of such patients, although they in fact constitute about two-thirds of the general nursing cases which have to be attended to in private work. Hence the training in these busy hospitals is apt to give the nurses the impression that only acute cases are deserving of special care. This accounts for the discontent of the private patient with the trained nurse so often expressed in ordinary cases. Yet the fault cannot properly be put down to the trained nurse for, after all, it is but the natural result of wrong methods of training.

How can these wrong methods be changed? Every nursing school which values its reputation in these days of higher education for nurses should in self-defence include in its scheme of training all departments of nursing, and so arrange the course that at the expiration of the first year an opportunity is afforded to each probationer to elect, if she choose, which branch of nursing she is desirous of following for a livelihood. The time has gone by when any training school of repute can remain indifferent to the future of its nurses. It follows that the managers of every school which is ambitious of success must make every effort to prepare the probationers who place their future in the training of their school, by arranging classes of nurses for hospital work, for special departments of nursing, and for nursing in private and district work. We must get rid of the feeling which generally prevails in many institutions that once a nurse leaves the hospital she must make her own way, and that it is no part of the responsibility of her school authorities to provide her with special instruction to this end. Another advantage of adapting the training to modern requirements would be, to widen the nurse's horizon and enlarge her sympathies. Too often the drill and routine, the exacting of implicit obedience to treatment regardless of the wishes and personal idiosyncracies of the patient, the importance of the various hospital physicians and the deference exacted by sisters, staff nurses, and others, all tend to give a probationer an exaggerated idea of the nurse's position, when the probationer finds herself free from hospital discipline, and placed amidst unaccustomed surroundings.

The modern nurse is then to a great extent the product of wrong and insufficient general training. It would not be just to lay all the fault on the nurse-training schools, for they are but the outcome of the demands for improved methods of nursing, and it is only within quite recent times that it has been possible, for the most experienced authorities, to adequately grasp the requirements which have to be met, and the dangers it is well to provide against in training. Now, however, that the causes of failure in many departments of nursing have made themselves definitely apparent, we have little doubt, that the necessary remedy will be promptly applied by the managers of every school, which is ambitious for its reputation, and genuinely anxious for the welfare and future success of its pupils. Each school which has a carefully thought-out curriculum, which affords each probationer adequate facilities to specially qualify herself in those branches of nursing which she may wish to follow on gaining her certificate, which takes care to warn its probationers against the fatal error of considering all treatment dubious, unless it is that to which they have been accustomed in their own school, will speedily abolish the wrong methods at present current, and secure, that every nurse, who has been trained in such a reformed school, may be found efficient and popular, wherever her lot may be cast.

### The Nursing of Infectious Diseases.

By F. J. WOOLLACOTT, M.A., M.D., B.S.Oxon, D.P.H., Senior Assistant Medical Officer, Park Hospital, Metropolitan Asylums Board, Hither Green.

LECTURE XX.—ENTERIC FEVER (*Concluded from p.* 63).

THE course of enteric fever is very frequently interrupted by the occurrence of complications, and indeed there is probably no other disease in which they are so numerous and so varied, for nearly every part and every organ of the body is liable to be attacked. Only the chief ones will be referred to here. The abdominal complications have for the most part been dealt with in the last lecture.

*Lung Complications.*—More or less bronchitis is nearly always present as part of the disease. Its chief symptom is cough, which, though not as a rule very troublesome, may at times prevent the patient from sleeping. In old people and in people with chronic affections of the lungs the bronchitis is apt to become severe and give rise to dangerous symptoms. Pneumonia and pleurisy sometimes occur and cause pain in the chest and increased rapidity of breathing. During the height of a severe attack, when the patient is much exhausted and the heart is growing very feeble, the circulation in the lungs may become very sluggish. The consequence is that the lower parts of the lungs become congested and waterlogged, so that very little air is able to enter. This is a very serious condition. The breathing is rapid and shallow, and, owing to deficient aeration of the blood, the face may become dusky and cyanosed. In nursing such a case it is of great importance not to allow the patient to lie on his back. He should be turned first on one side and then on the other, the position being changed every few hours; and, if necessary, he should be supported by pillows to prevent him from slipping back. Our object in this is to drain away, as far as possible, the congested blood from each lung in turn, and so to enable it to fill with air.

Occasionally the larynx becomes inflamed and perhaps ulcerated. The symptons are croup and difficulty in breathing.

*Boils* and small abscesses under the skin are comparatively common, especially after the cold bath treatment. They occur chiefly on the back and in parts subjected to pressure. They may be treated by the application of boracic fomentations, and they should, as far as possible, be protected from irritation and pressure.

*Parotitis*, or inflammation of the parotid gland. This gland is situated at the side of the face, below the ear, and is one of the glands supplying the mouth with saliva. When inflamed it gives rise to a swelling extending forwards on to the cheek, and as a rule, sooner or later, breaks down into a number of small abscesses.

*Phlebitis* or inflammation of a vein and clotting of the blood in it. Usually it is one of the veins of the thigh or leg that is affected, the symptoms being pain, tenderness and swelling, with some rise of temperature. In the treatment complete rest in bed is essential, with elevation of [the limb. The tender parts may be painted with glycerine of belladonna, and covered with a large fomentation, and a bandage carefully applied from the toes upwards. The patient should not exert himself in any way, for any sudden effort or straining might loosen some of the clot in the vein and set it free in the circulation. If such an accident happened sudden death might result. For the same reason the limb should always be very gently handled and never rubbed.

*Bone and Joint Inflammations.*—The symptoms are pain, tenderness and swelling. The inflammation of the bone usually goes on to the formation of pus, and extensive mischief may result.

*Cancrum oris* or gangrene of the lips and cheeks is rare and occurs chiefly in unhealthy and neglected children. The appearances and treatment have been described in the lecture on Measles.

A curious complication sometimes observed is known as *Tender Toes*, the toes and feet being so tender that the slightest pressure causes pain. It is most common after the cold bath treatment. A cradle should be placed over the feet to take off the pressure of the bedclothes.

If the patient be pregnant, and the attack of enteric fever be at all severe, premature labour or abortion is likely to take place. In such cases there is, of course, considerable danger, but with care both mother and child may do well.

*Complications during Convalescence.*—Some of the complications already considered may arise during convalescence. Among these are bed-sores, and inflammation of a bone or a vein. After the patient has been allowed to leave his bed it is not uncommon to find that much weakness still remains in the legs, and that they become swollen if he walks about, while in some cases small hæmorrhagic spots appear on the skin. These symptoms soon clear up as the strength is regained. Loss of hair and temporary baldness is sometimes seen.

Occasionally the mental |condition undergoes a change. There may be loss of memory and feebleness of intelligence, and in rare cases acute mania may appear.

*Treatment during Convalescence.*—The precautions and care necessary during the acute stage must be continued till the patient is well advanced in convalescence, and the possibility of a relapse should be borne in mind. After the temperature has fallen to normal he is often left very feeble and wasted. He must remain quietly lying in bed for some time longer, and the precautions against bed-sores must not be relaxed. The food is cautiously increased, and care must be taken that nothing is eaten but what is expressly ordered. Some watchfulness will be necessary, for the patient is often extremely hungry, and eager for anything that injudicious friends may supply him with. As he grows stronger he is allowed to sit up in bed, supported by a bed-rest, and then gradually passes through the stages of getting up, dressing and taking exercise.

*After consequences.*—After enteric fever many months may elapse before the patient regains his usual health and strength; but, as a rule, he will in time make a complete recovery. In some cases, however, permanent bodily or mental weakness may result, and any of the organs affected by complications may be irretrievably damaged.

*Precautions against Spread of Infection.*—The infection is almost entirely given off in the discharges, especially the stools, the urine, the expectoration, and the pus from abscesses, particularly those in connection with diseased bone. All these discharges, and any soiled articles of clothing must, therefore, be very cautiously handled. It is particularly important to remember that the urine is often intensely virulent, indeed, in many cases, much more so than the stools. Soiled garments and bedclothes must always be removed at once, and all the precautions described in Lecture III. of this series must be carefully observed. If the discharges are allowed to dry on the clothing before removal, minute portions of them might be accidentally brushed off in the form of dust, and so escape into the air, and perhaps be swallowed or inhaled. This is probably one of the chief ways in which the infection spreads. The practice in dealing with the stools and urine varies considerably, and the nurse will, of course, be guided by the directions of the doctor for whom she is working. To ensure their complete disinfection it is necessary to burn or boil them, or to add at least an equal bulk of some strong chemical disinfectant, such as $\frac{1}{20}$ carbolic acid solution, or $\frac{1}{10}$ lysol solution. After thorough stirring, the mixture must be covered up, and kept for at least half an hour before being emptied into the drains. The expectoration, and any dressings from abscesses should be burnt, and bed-pans and all other utensils kept scrupulously clean.

The nurse should take special pains to keep her dress from being soiled with surcharges, and in changing the patient she should tuck up her sleeves above the elbow. The forearms and hands should be carefully washed, especially before meals, the nails being kept short and the use of the nail-brush not omitted. After washing, it is a good plan to immerse the hands for a short time in some disinfectant solution, such as $\frac{1}{1000}$ corrosive sublimate, and then to thoroughly rinse them in clean water.

In general hospitals it is the custom for enteric fever patients to be nursed in wards side by side with other cases. It is then essential that all utensils, feeders, bed-pans, enema syringes, etc., used by such patients should be reserved entirely for them alone; and a conspicuous mark should be attached to each, so that no mistake is possible. A special thermometer should be used. A useful device is to fix some prominent sign to the head of the bed, so that the infectiousness of the disease may never be lost sight of.

*Summary of Nurses' Duties in Enteric Fever.*—The temperature is to be taken, and the pulse and respiration counted every three or four hours, as directed, and the results entered on the chart. All this is to be repeated half an hour after any measures have been taken to reduce the temperature, so that the effect may be correctly estimated. The patient and his clothing must be kept scrupulously clean, the mouth frequently cleansed, and precautions taken to prevent bed-sores. Food is to be given at regular intervals. The position of the patient in bed must be from time to time altered if necessary. Precautions must be taken to prevent the spread of infection. The nurse should take pains to find out the wishes of the medical man on any doubtful points. This is especially important in enteric fever, for the treatment adopted by different men may vary widely in nearly every particular.

The nurse's report should ontain information on the following points: (1) The number and character of the stools. (2) Whether urine has been passed or not—retention or incontinence. (3) The amount of food taken, and any special liking or dislike of the patient. (4) The amount of sleep, and its character. (5) Presence or absence of delirium or restlessness. (6) Any complaint of pain, or discomfort of any sort. (7) Any unusual symptom. (8) Any complication or other change.

The nurse should be on the alert for the onset of complications, especially the more serious ones, such as hæmorrhage or perforation. The symptoms of these two have been previously described, and when they appear information should be sent without delay to the medical man. The patient should be kept under close observation throughout the illness, for even slight changes may be important; and as far as possible, this should be done without attracting his attention, and without any fussiness. After having been off duty the nurse should take pains to find out what has happened during her absence, and should carefully read the report of her colleague. When all this has been done, and done well, one thing more is necessary. The nurse must try to secure the confidence and trust of her patient, and if she is gentle, cheerful and sympathetic she will be of more real help to him than can easily be estimated.

### The Nurses' Clinic.

### SO-CALLED "WELL-CASES."

Those who have taken up the private nursing of children will come across cases which they are in the habit of styling "well-cases." Some nurses object to going to this kind of case. They say that it is as unsuitable for a trained nurse as it is unpleasant. They think it sheer waste of valuable time and energy, and would like all such applications to be rejected and themselves sent only where definite sick-nursing is required.

Now, are they right; and is such work undesirable and unprofitable for trained nurses?

First let us consider what these cases are. To begin with, there is the large class of those babies who are being mismanaged, whose diet is not agreeing, who are giving unusual trouble over teething, or perhaps are being so spoilt as to develop habits and ways that would hardly be credited unless seen. There are the very wealthy, a class for whom the private nurse must always be prepared to reckon, as they will often apply for her services without due cause, and may even wish her to undertake the entire upbringing of a perfectly healthy child. There is the nursery left suddenly in the lurch by the illness or gross failure in her duties of its ordinary head. And finally there is the large class of those cases where the doctor in charge himself sends for a nurse, to practically place the matter in her hands, with or without a few directions and an occasional visit.

Now many of these cases are very ill-defined. It is impossible to judge of their merits until the nurse has actually taken up her duties, and therefore it seems that she must at least be prepared to go to them. Inexperienced parents are nervous and easily alarmed. In some instances, of course, it is not even pretended that the child or children are other than healthy, a suitable children's nurse is all that is required; but, as one cannot always be found at once, there may be a very real emergency. And in many instances the child turns out to be very delicate, or suffering from some congenital defect or weakness, or so badly mismanaged as to cause serious loss of health.

In most of these cases, therefore, a trained nurse may be of as much real use as in acute illness. The work may not be as interesting, indeed hardly can be to one who loves her profession. It may be somewhat monotonous, and involve some loss of experience, but even so it may be as well worth doing. Personally, at any rate, I have found it so. I have been private nursing, chiefly among children, for nearly six years, and have had many of these so-called well-cases. I do not like going to them, and have looked upon them as a necessary, rather than an agreeable, part of one's duties, but many of them have proved very satisfactory, and many are very short. One tiny baby I had, wizened and wailing, who was simply being starved; it required the strength of its food doubled, and in a week was well, fat and happy. Several other cases only lasted a fortnight—in one the people only needed reassuring; another was to give the permanent nurse a holiday; a third was to break a boy of over two years of taking all his nourishment through bottles, a task that had been tried so often without success that the child had absolutely to be allowed to starve himself on a few mouthfuls of biscuit and no drink for 30 hours before he would give in, after which there was no more difficulty, as, fortunately, his power of digesting the proper diet for his age was unimpaired. A longer case was that of two baby

THE NURSES' CLINIC—*Continued.*

girls whose mother had deserted them, inducing their nurse to do likewise without notice. There were considerable difficulties to contend with here, though none with the father or children, and I have always been thankful that I was able to leave those worse than motherless babes with a truly superior and good woman, who is bringing them up well Another time I went to a boy of 19 months who possessed only four teeth and showed no signs of others coming. Towards the end of my stay he cut nine, and the remaining seven were well on their way. And many other cases proved to be more really delicate or ill than was supposed. One in particular, a boy of nearly two, turned out a very unusual case of malassimilation. He was a veritable skeleton, tall, with most of his teeth, and weighing 15 lbs.! It took four months under the directions of the best doctors to make much impression here, and after all the poor little fellow died in another three years. But at any rate, from the most utterly miserable of babies to be found, in hospital or out, he became in a few weeks a supremely happy one, and so remained for the rest of his short life.

But it is not necessary to multiply instances. I have quoted these to demonstrate my point, that many of these cases are quite suitable for a trained nurse. Of course, there are exceptions, but these with tact and plain dealing can nearly always be made very short. Surely on no staff would a nurse be required to stay where she was not really needed, or longer at any one such case than two or three months. Still less would pressure be put upon her against her desire to undertake the upbringing of a baby.

Finally, a few words as to her position at such a case, which is one usually filled by the servant class, and therefore not so well-defined and easy even among the nicest people as that of a nurse in charge of a sick-room. But let her be simple and straightforward in her dealings; above all, not fancy that she is demeaning herself; and I think most of the difficulties will vanish. One of the biggest often is the question of pushing a perambulator. Personally, I do not object to doing so, or consider it, even in London, beneath my dignity. But I am not a great walker, and find it very tiring. There is generally night-work at these cases, and as one must be fairly fresh to fulfil one's higher duties—I say so plainly—and have nearly always been relieved in some way of a good part of the necessary "pram-work." Let a nurse state quietly and simply what she is able and willing to do, and she will usually find that people are quite ready to make arrangements accordingly. Still, of course, much depends upon the household she is in. She must not expect impossibilities. If, in fact, as is so often said, she is not prepared to be "all things to all men" as far as in her lies, she should not undertake private work.

### District Nursing in Prussia.

BY AN ENGLISH NURSE.

Last year my hopes of some years were at last realised, and I was given a district. I own at first I was a little disappointed when I heard that my new work was right in the country, but now I feel that I love my district. What joy that memorable Tuesday when I found myself and my boxes really safe at the station in Schwerin, and knew my great wish was at last about to be realised! Yet, when the supreme moment came for saying good-bye to the nurses with whom I had been working for nearly four years, how sad I felt! Presently the train steamed into the station, and the committee-lady of our Red Cross Society, who was to introduce me to my new committee, got in with me and we started off on our journey from Mecklenburg into Prussia. Now, indeed, I felt lonely and home-sick, though it was only bidding farewell for a time, as our committee likes all the nurses not in the Mutterhaus (mother-house) itself to spend a few days under that roof at least once a year, so as to keep in touch with the other nurses and not forget the rules, etc. Going from Mecklenburg into Prussia is the same thing as an English girl going to Scotland or *vice versâ*, and it seemed strange as the train passed out of Ludwigslust, the last Mecklenburg station, to note that instead of the red, yellow, and blue colour of Mecklenburg there was only the black and white of Prussia. The folk, too, looked different at the stations, and later on I found the manners and speech of the people and the ways of building the villages all different. When we arrived at the station we found a smart carriage and pair waiting for us, and very soon were quickly on our way to my new destination, through very flat but pretty country, with lovely forest and meadows. At last we drove down a splendid avenue of fine chestnut trees (very characteristic of Prussian estates in the Prignitz, Alt-mark, etc.), with a nice old castle in front of us, and a few seconds later we rattled over a stone bridge, across the broad moat, into the courtyard, where the owners were waiting to receive us. After a very warm welcome and light refreshments we came over to the steward's house, about eight minutes' walk from the castle, and I was shown my rooms. The bedroom was fresh and clean, all white, with a pretty text over the bed and a beautiful real country bunch of flowers on the dressing-table. The sitting-room, too, was pretty in the sunshine; it is very large, and at first it seemed too large, but now, with the piano the owners of the castle have lent me, I love it as it is. On that particular day I remember there were such lovely tea-roses and beautiful carnations all about the room that I felt happy at once, though I did not then know how happy I should become here.

### My First Patients.

Then came the hour for parting with our committee-lady, whom we all love, but I had no chance of being lonely, as my kind hostess took me to the castle for the evening. Since then, fortunately, I have had no time for home-sickness and sentimentality, for the very next morning I discovered an old woman on the estate with dropsy, and with terrible running sores on both legs which took time to dress morning and evening, and the next week the gardener's wife had to undergo a serious operation. Then a child's thumb had to be amputated, and there were several other serious cases, congestion of the lungs, etc. I have altogether four parishes, including their villages, which I visit at least every week, and of course, where illness is, daily, or as often as possible; so I am fully occupied. Some of the villages are too far off for me to visit daily, either driving or bicycling, for the district is as large as Surrey, and there are only two nurses as yet—one of our Red Cross nurses and myself. The distance, too, between the villages is considerable, and the roads so bad that it is impossible to get at them; so that when the need is great we have to go and stay. In November I was three weeks at a large village on the Elbe. The patient was a very, very poor woman from East Prussia, with child-bed fever. There was great poverty there, so much so that the first thing I did was to beg for sheets, underlinen, etc., for the woman. The neighbours were really very kind, and gave gladly and willingly, so that every day different families brought milk, eggs, meat, etc., so that at least I had something to cook with. I myself lodged at the clergyman's house, and used to go to my patient from there. After three weeks I was able to return to my beloved S——. Oh, how glad I was to get back! How nice the welcome at the station, when Frau von V—— (the owner of the castle) met me and drove me here herself, and then the lovely chrysanthemums I found waiting for me, together with a beautiful picture of Christ and Mary and Martha, which I had long wanted. My patient is now quite well. I met the doctor yesterday, who told me she is even back at work again. The whole Christmas week I was at the castle, doing my work from there, and we all had a very merry time.

Bicycling under Difficulties.

Now, after a hard fight, I have a bicycle. I once thought that being able to ride in London would make me able to ride anywhere. Now I think differently, for here the roads (if one may call the ways for carts and horses "roads") are almost impassable in the winter; and if very dry in summer, too, I often have to "bike" on a path not a foot wide, and with a deep ditch on one side and very deep cart-ruts on the other, and so uneven that I am nearly jolted from the saddle. However, practice makes perfect, and I get about now without jumping off and walking most of the way, as I did at first. But even now bicycling still has many drawbacks. The other day I had to go to a village about 5½ miles away, and was really very busy, so had no time to walk. It had been snowing all the morning, and was disagreeable weather. At first all went well till I came to a meadow, which is always wet. This time it was quite under snow, and I pushed my bicycle as far as I knew in the right path, till suddenly crack! crack!—bicycle and I were through the ice into the water. That was the beginning of my many troubles, for I found the plank of wood forming the bridge so slippery that I nearly fell into the little stream running through the meadows. I found the next plank broken; however, not wanting to go all the way back, and being obliged to get over, there was nothing for it but to wade across in the ice-cold water, and of course all this delayed me too. Sometimes I have to go through the forest. In some parts this is all right, but in others one has nearly to double up under the overhanging branches, and to exercise great care for fear of running into the trees.

Outside Work.

Not only does a Gemeinde schwester have nursing to do, but if her time allows it she tries to get the people interested in each other, etc. Thus every fortnight, from 7 till 9 p.m., all the women on the estate bring their knitting, etc., and come to my room. We start with a hymn, then talk; I read aloud, we talk again; then I play their popular songs, as the Lorelei, etc., and they always sing very heartily; then I read prayers, we sing a hymn, and all is over. The people appear to like it, for others, from two neighbouring villages, asked if they might come too. On Sundays I have a Sunday-school class, girls from two to three, boys from three to four—that is, if I have no patient to go to then; but as yet I have only been obliged to miss twice. Of course, one has scarcely a minute to oneself, but that is nice where one is right in the country—one hour's quick drive from the nearest station, and two hours' from the nearest town. On this estate the owners are very good and kind, and take much interest in their people; but in the villages, where the peasants feel no one cares for them, they become hardened, and, instead of being interested in their work and land and feeding a few pigs, etc., they sell their potatoes, etc., and, of course, grow poorer and poorer, take to drink, and get very dirty, going about with their clothes in rags. Perhaps in time they may improve; at present it is uphill work.

### Some Curious Encounters in Nigeria.

BY AN OLD GUY'S NURSE.

Some of my experiences since I commenced work in the Government Hospitals in Nigeria are rather out of the ordinary run of a nurse's life, and may be interesting to your readers.

It is certainly rather a novel "experience" to be chased by an ostrich on the way home from a tennis party, but such was my lot on one occasion. The bird was considered a tame one, and was kept as a pet by the Governor, but it had been greatly excited by the barking of a dog, and having got loose, on catching sight of the little party of nurses and members of the Governor's staff who were escorting them back to the hospital, proceeded to give chase. The party scattered, and vainly attempted for some time to elude the bird. A small hillock had to be skirted, and by darting round to the other side they hoped to get out of his sight. But he was too sharp for them, and on he came. I tried to jump a ditch and lost my footing, with the ostrich close upon me. Fortunately, there was a railing in the bird's way in which he caught his neck, and before he could extricate himself I and my friends made off as quickly as possible.

This little adventure was not the last for that day however. The hospital is built on an island in the river, and as we were being rowed across by the native boatmen we had the horrible experience of feeling the boat being violently jerked underneath, and knew that it could be caused by nothing else but a crocodile. The native was paralysed with terror, and would willingly have left us to get to land as best we could; but as there was no way of escape except into the monster's jaws he was obliged to continue rowing, and thus brought us safely to shore.

On the voyage home I was in charge with another sister of a patient who was very ill with a fever peculiar to the tropics. I was on night duty, and one night I was watching my patient, about whom I had been very anxious for days, and was relieved to find him sinking into a refreshing natural sleep—the first time since the fever began. Suddenly there was a shock and vibration through the vessel. All was commotion. Someone coming along warned me to put the life belts upon my patient and myself, as there had been a collision with a German vessel. I was dismayed, and reaching for the life belts the thought flashed into my mind, "What a pity to disturb him when he is in such a nice sleep." Even as I stood by him with the life belt in my hand he opened his eyes. I immediately dropped it on the floor and drew a rug over it, lest the sight of it should alarm him. Even as I did so I heard a step outside, and opening the door I experienced intense relief and satisfaction upon learning that the danger was passed. The patient slept again, unconscious of evil, and awoke in the morning with the fever gone. As the steward was relieving me in the morning he told him of what had happened in the night, and when I returned he inquired "why had I not told him of what was going on?" I said that "I never intended him to know." "Well," he rejoined, "I woke once in the night and saw that you were looking uncommonly white, and I would have asked you to go and lie down—as I thought you must be very tired—but I knew it would be of use, as you never will!"

The sisters in Nigeria always welcome The Hospital.

### Visit to a Chinese Native Hospital.

BY A SPECIAL CORRESPONDENT.

It may be of interest to your readers to learn how a purely Chinese hospital is managed, and the patients nursed. A good deal has been written at times in their annual reports by medical missionaries giving their experiences in the hospitals, established by various missions in China, but these do not show how the natives run their own hospitals, and hence some particulars of the Tung Wah Hospital, in Hongkong, which is managed exclusively by natives, may prove acceptable. I may mention that though the Chinese as a race are singularly callous to suffering, there is, in the larger cities of China at all events, generally a hospital or institution for the relief of the sick, and occasionally an asylum for the blind, while the shopkeepers contribute, it is to be feared under some pressure from the Beggars' Guilds, daily *cash* (the smallest Chinese coin) towards the maintenance of the lame, the halt, and the blind, and, let me not forget to add, the leper. Not infrequently the hospital committees enlarge their sphere by ministering to the relief of those suffering from famine or other sudden calamity, as is notably done by the Oi Yuk Tong in Canton. In Hongkong the Tung Wah Hospital Committee, elected yearly by the Chinese community, charge themselves also with the care of the Chinese paupers as well as the sick, and consequently wield considerable power over their countrymen in the colony. The committee are, however, in some degree under the control of the Government, and about ten years ago, during the administration of Sir William Robinson, a successful effort was made to bring the hospital more into conformity with the requirements of decency and civilisation. Prior to that time dirt and disorder reigned triumphant, and the crudest Chinese methods only were in vogue in the hospital, which was, in fact, more of a mortuary and poorhouse than an institution for the healing of the sick. Now. however, patients can take their choice of purely Chinese treatment or treatment by Chinese doctors trained in Western methods. The latter are, moreover, under inspection by one of the Government medical staff, and the hospital is included in their rounds by the justices, who once a week visit the hospitals of the colony. But, as I have said, it is managed solely by Chinese, and, as now conducted, is a credit to the committee.

### The Hospital.

The Tung Wah buildings—for there are three blocks—are situated in the western district of the city of Victoria, not far from its great central artery, Queen's Road. The entrance is through a large door in a massive wall, ornamented by fanciful openings framed in green glazed tiles, and the visitor is at once ushered by the smiling Chinese doctor in charge into the great hall, where the committee hold their solemn conclaves. This is a really fine chamber built in purely Chinese style, with the roof supported by handsome carved teak pillars, and the floor paved with Chinese grey marble. It is furnished entirely with carved blackwood chairs and tables, and is adorned with gilded extracts from the Chinese classics. The hospital was erected in the early seventies, and it has since been enlarged more than once. From the hall the visitor is conducted through the various wards and finds the larger number of patients suffering from phthisis and beri-beri, the latter disease being especially common, so much so that sometimes nearly half the cases are credited to it.

### The Nursing.

The attendants are all men except in the female wards, where native women act as nurses. It does not appear that either have received any special training or displayed any great aptitude for the important duties they undertake. They supply the wants of the patients, which are commonly of the fewest, administer the medicines (many of which are strange to the Western pharmacopœia), and no doubt apply the plasters and bandages, which in Chinese practice take the place of surgery. In accident cases—unless the Western-trained doctor is consulted—it is a matter of chance whether the patient pulls through; more frequently he either dies or leaves the hospital with some deformity which he will carry to his grave. It is worthy of note, however, that increasing numbers of the Chinese now submit to necessary operations and usually make a good recovery. The wards, which in former times were allowed to become most offensive, are now kept clean, and plenty of fresh air is admitted, while crowding is no longer permitted. Of luxuries or cheerful surroundings there is no pretence. The sole furniture consists of the beds, made of trestles on which boards are laid, red blankets and Chinese pillows—hard leather boxes. The patients are allowed, when sufficiently recovered, to sit or lounge about on the spacious verandahs which, as in all Asiatic hospitals, extend the whole length of each floor. Some of them are so lively that it is at first difficult to believe that they have anything the matter with them, especially sufferers from beri-beri, who appear, in the early stages of the disease, to feel little pain. In the women's ward there are frequently several quite young girls suffering from this disease, and as many as 130 beri-beri patients in all.

### Chinese Stoicism.

The Chinese appear to bear pain with considerable stoicism. As a matter of fact they do not, I am told, feel operations so keenly as Europeans, and the wounds made by the surgeon's knife heal much more rapidly with the Chinese. I remember seeing two Chinamen in the Government Civil Hospital who had just undergone operations for injury to their legs by an accident on the Peak Tramway and in one case amputation had been necessary; they were quite cheerful and comfortable, and the one-legged man was soon able to make trial of the wooden substitute which was supplied to him on his convalescence. In many minor operations the Chinese often decline to submit to an anæsthetic.

### The Annexe.

The annexe to the Tung Wah is quite a fine structure, more Western in its architecture than the original building, and the wards are lighter and more cheerful. The children are all lodged in this block. There is, however, no superfluity of furniture, and the nursing is the same as in the parent institution. It is situated on the opposite side of the street, and was built by subscription, the Government granting the site. It has only been open about eighteen months. On the ground floor there is a spacious hall, adorned with a full length portrait in oils of Governor Sir Henry Blake, who laid the foundation-stone. The addition of this block to the institution marks a distinct advance, and it is expected that, in the course of a few more years, the committee will see their way to not only tolerate, as at present, Western methods in the hospital, but be eager to encourage their general use.

### The College of Medicine for Chinese.

In conclusion, I should mention that the way for this important reformation is being prepared by the Alice Memorial Hospital, in which Western methods have always been pursued, and with which is affiliated the Hongkong College of Medicine for Chinese. This latter institution grants diplomas to the graduates, and many of them have turned out very creditable assistants to the European doctors. One of these licentiates presides over the ward in the Tung Wah Hospital, in which Western medical treatment is adopted. The prejudice against Western doctors is still strong and widespread amongst the Chinese, so much so that, when shockingly injured by an accident and amputation is the only alternative to death, they will more often than not resolutely refuse to be placed under Western treatment. This prejudice, like all Chinese prejudices, will melt very slowly, even in Hongkong, but it is gradually becoming less pronounced.

### Nursing amongst the Ziguas.

BY A CORRESPONDENT.

The temperature is 90 degrees in the shade on the deep verandah, where the lizards run up and down the wall, and the cicala's shrill call is intermingled with the incessant humming of the Carpenter bees. Far away in the distance rises the smoke of a huge fire, and the fleecy clouds just fleck the mountain tops.

We are looking across the vast Zigua plain that stretches out from the foot of the mountains, which run down from the westward, and over the Pangani, a river fed from Kilimanjaro.

Here we have a medical station, and treat the natives and Europeans—such as there are—for their varied complaints. Until quite recently there were no others here besides Ziguas but the advance of civilisation in the form of a railway has brought up a heterogeneous population of Orientals and others.

The ubiquitous Greek is present, and is a person here of some importance, being the builder and hotel-keeper and general storeman of the place.

The Hindus are low caste. Zanzibar is said to have the sweepings of India, and we have here apparently the sweepings of Zanzibar. The native town of Korogwe itself is a most characteristic one. Built on an island, where the river forks, it is only accessible by narrow bridges—a relic of the not very far distant days of Masai raids—it is a highly defensible stronghold, as the water runs in a rapid and swirling torrent difficult to cross, and alarming even to the view of the inexperienced traveller who ventures upon the frail structures of tree-trunk and creeper which span it. The houses are beehive shaped, and have no exit for the smoke beyond that afforded by the thatch. It is a plan not altogether without its advantages, seeing that smoke is an enemy to the mosquito. Round the huts tumble and play the fat brown little babies, with their protuberant abdomens, which reveal too many big spleens for the climate to be wholesome to a European after dark.

### The Work on the Station.

Our station stands on the side of Fundi Hill, and though we have no hospital yet there are sufficient sick people to keep a single nurse busy. A doctor, who had been wanted for a long time, having just arrived, and having operated upon various cases, has increased business very considerably; and the area of work, instead of being limited by the compact ward of a hospital, is extended over a distance of nearly half a mile. Behind the house stands a hut built of wattle and daub, floored with hard beaten mud, where some of the schoolboys sleep. Lately it has been used for a sick room as well, for the disease which has been the plague of the country has attacked our boys also. This is none other than the ordinary measles, but, as so often the case in savage countries, it proves very fatal to young children. Eye troubles are exceedingly common after it, and in consequence there is a native belief that if children go to sleep they will lose their vision. This not unnaturally adds considerably to the mortality, as the mothers perpetually jog them when nursing, in order to keep them awake, until they are tired out, and sometimes die from sheer exhaustion. However, the parents are gradually being educated into better ways. Among the boys on the station, thanks to care and attention, there have been no evil results beyond a little conjunctivitis.

### Crocodile and Lion Bites.

In a hut next to the one in which are the boys lies a stalwart Zigua, the victim of a bite from a crocodile, his arm broken in two places, and suffering from a condition with which it is rather difficult to deal, especially when resources and suggestions are not very abundant. Some of these Africans are wonderful patients and stand pain with the most marvellous fortitude, provided they have faith in their doctor. Crocodile and lion bites are both of them said to be very septic, and consequently it is a matter of some anxiety as to how this man will progress. He is an important personage, being a churchwarden. On the slope of the hill that stretches from the mission down to the church are placed yet more huts, and in these are quite a remarkable selection of cases—cataract, an injured arm, a leper, and a bad case of tertiary syphilis. The huts are all under the charge of one nurse, and in addition she has an out-patient department where sores, coughs, cuts, and other small ailments have to be attended to. Her only permanent help is from a small boy whom she is training. Add to this the care of any sick European who happens to be passing, and it will be seen that her lot is by no means a light one.

### The Blacks and Fatalism.

Amongst other patients who were treated here recently, were some of the Boers who were trekking up country. It was just at the time that the railway was being built, and the barrow pits were full of water after the rains. Mosquitoes and blackwater fever were rife, and several fell victims to this deadly disease. It is not nearly so prevalent now since the railway has been completed, especially during the dry season, but has followed the line further up country, where the next station is being completed. In nursing black folk operations as a rule do well. But although the patients show good recuperative powers, yet should they lose heart and think they are going to die, they will probably do so, in spite of every effort made on their behalf.

### To Nurses.

We invite contributions from any of our readers, and shall be glad to pay for "Notes on News from the Nursing World," or for articles describing nursing experiences at home or abroad dealing with any nursing question from an original point of view according to length. The minimum payment is 5s. Contributions on topical subjects are specially welcome. Notices of appointments, letters, entertainments, presentations, and deaths are not paid for, but we are always glad to receive them. All rejected manuscripts are returned in due course, and all payments for manuscripts used are made as early as possible after the beginning of each quarter.

### The Nursing Question in Bordeaux.

At a time when the standard of nursing in Paris is being gradually raised, it is due to Bordeaux to recognise that a start in the right direction was made in that city as long ago as 1884. But of the numerous ladies who attended the School for Nurses at the Protestant Hospital, although many successfully passed their examinations, the majority refused to become practical assistants in the wards, so that the hospitals in no way benefited. Three years back, however, it was determined only to receive probationers who would become so in real earnest, not only passing their examinations, but entering the wards and working in them.

There has too been a further striking development in the establishment of a School for Nurses at the Civil Hospital du Tondu, at Bordeaux. This scheme was started first in the immense hospital of Saint André, where the poor were tended by religious sisters as well as by lay nurses, and where it was hoped that all indoor probationers, outdoor probationers, sisters of St. Vincent de Paul or of Nevers, Roman Catholics and Protestants alike, could be taught together. It is not surprising to learn that this attempt failed, and, after considerable trouble, it was determined that a new school should be established at Hospital du Tondu with 120 beds, on much the same lines as that of an English hospital. The greatest difficulty was the choice of a suitable matron. No French nurse seemed capable of fulfilling satisfactorily such a position, because of necessity she lacked the experience of the many-sided duties required of her. Therefore the authorities decided to offer the post to a Dutch lady; but, partly owing to her endeavours to run the establishment entirely upon Dutch lines and the trouble which she herself experienced in mastering the language, this first venture did not prove a success, and after three months' trial an invitation was made to Miss Catherine Elston to come to the rescue. She had trained at the London Hospital, and had been nurse at the Poplar Accident Hospital, besides having seen service under Mlle. Hamilton at the Protestant Hospital, Bordeaux.

Then, at last the authorities felt that they would be able to offer probationers not only such theoretical training as had before been thought sufficient, but also a practical course of instruction under a capable teacher. To enter this school the probationers must be between the ages of 18 and 35, must have received a good education, be in the enjoyment of good health, and of thoroughly good moral character. They have assigned to them pretty little rooms, somewhat of the nature of bed-sitting-rooms, nicely appointed, with furniture of polished pitch pine. Those who desire society and recreation have the use of a large nurses' sitting-room, where music and singing are allowed. There is also a nurses' dining-room where the probationers take their meals in two relays. Here conversation is freely allowed, and the directress is usually present.

The probationers are both free and paying. The former obtain admission upon the understanding that they will remain as salaried nurses in the hospital for two years after they obtain their certificate. The paying probationers are under no such obligation, but they have to pay 1,000 francs (£40) for their two years of study, which includes board and lodging. During the first year they are taught the cleaning, sweeping, and polishing of the wards, the preparation of baths, the making of beds, and the superintendence of the patients' meals. The second year they still do a great deal of the domestic work of the wards, but they are also allowed, if capable, to assist the nurses and sisters. At the end of the two years if they obtain their certificate, they assume a greater measure of responsibility, but still hardly so much as falls to the share of a three years' probationer in most English hospitals. The sisters undertake much the same duties as the sisters at home.

With regard to instruction, lectures of one hour are given each week during eight months of the year to the probationers, and these include the elements of anatomy, physiology, hygiene, and pharmacy, small operations, the nursing of women and children, of surgical and medical patients, and the initial rules of hospital administration. The probationers wear pale blue linen dresses with white caps, aprons and cuffs, and for outdoor uniform a black velvet bonnet with white strings and a long cloak.

It is the boast of the directors of the Tondu Hospital that it is still the only school in France where nurses can be taught in a civil hospital under conditions which ensure success, and the visit which was recently paid to the school by M. Casimir-Périer, during his short stay in the town, has done much to encourage the promoters.

### Incidents in a Nurse's Life.

**Contributions for this column should be addressed to the Editor, and if accepted will be paid for.**

### A COTTAGE HOSPITAL EXPERIENCE.

I shall not soon forge t my first experience of hospita festivities after being appointed matron of a cottage hospital of 16 beds.

The secretary approached me on the subject, telling me it was their custom to give a yearly entertainment in one of the wards to which old patients were invited. The hospital was comparatively new, and he considered that it roused interest in the town and neighbourhood to invite old patients to a festive gathering.

I demurred to his suggestion, as the work had much increased during the past year, and it seemed to me very doubtful whether at any time all the patients in either male or female ward would be fit to be turned out of their beds, which would have to be taken down for the occasion, also my nurses were few.

My objections, however, were overruled, and preparations went forward, my share being to arrange for the feeding of the visitors, about 100 in number.

Two days before the event a very bad accident was brought into the male ward—a collier whose spine had been crushed by a fall of coal. I therefore decided that the entertainment must be held in the women's ward.

The night before the event, about 9 P.M., a doctor telephoned that he was sending in a child with acute intestinal obstruction for immediate operation, and at once we were plunged in the work of preparation. When the operation was over we moved the little girl into a tiny isolation ward adjoining the operating-room, and I stayed up with her until 4 A.M. as I had only one night nurse and the child could not be left for a moment. A day nurse then relieved me and I crept wearily to bed wondering how I should get through the next day's duties with this additional work and anxiety. As I was running downstairs in the morning to our eight o'clock breakfast the telephone bell rang and I heard to my dismay that an old man was being sent in who had had his leg smashed whilst working in a colliery. He arrived in a collapsed condition and we thought that he would scarcely live through the operation of amputation of the leg.

The morning was half over by the time the old man was brought from the operating-room to bed, and then the rush of the day began. I had only two day nurses, one of whom had been up half the night. The child operated upon the previous evening was dying and could not be left. The old man had to have constant watching; the ordinary routine work—housekeeping, dressings, patients' meals had to be done; the beds in the women's ward had to be taken down and the patients made as comfortable as possible, some of whom became hysterical in their excitement; bread and butter and sandwiches had to be cut in large quantities and all preparations made for feeding our visitors.

In spite of all difficulties we were ready at the hour, and everything passed off satisfactorily, the entertainment—partly musical, partly dramatic—being much appreciated.

By midnight the ward was in order once more and the worn-out matron and nurses were able to retire to their beds, a special nurse having arrived for the child, who died during the night. I am glad to say that the old man made a good recovery.

Since then I have resolutely turned a deaf ear to all suggestions of entertainments on a large scale, which would necessitate dismantling an occupied ward or involve excessive labour to nurses already sufficiently worked.

### Central Midwives Board.

A meeting of the Central Midwives Board was held at the Board-room, 6 Suffolk Street, on Thursday last week. There were present:—Dr. Champneys, Dr. Dakin, Mr. Fordham, Mrs. Latter, Miss R. Paget, Sir W. Sinclair, and Mr. Parker Young.

The first business on the agenda was the election, by ballot, of the chairman for the ensuing year, Dr. Champneys being unanimously voted to that office (Mr. Parker Young in the chair). Amongst the correspondence before the Board was a letter from Dr. Kaye, County Medical Officer of the West Riding of Yorkshire, inquiring on behalf of a Conference of county medical officers whether the printing of the Roll of Midwives could be arranged according to geographical areas, and whether it would be compulsory for all midwives to carry the entire list of articles enumerated in Rule E2. It was agreed to make a note of the suggestion as to the Roll, and, with regard to the second question, that the Board had no power to make exceptions to the rules. A letter from Dr. Niven, M.O.H., Manchester, was read, asking as to the meaning to be attached to the word malpractice in Section 8 (2) of the Act. It was agreed to reply that each case must be judged upon its merits.

### The Number of Examinations.

Memorials were presented from the committees of the British, General, and City of London Lying-in Hospitals, with a letter from Queen Charlotte's, asking that the examinations of the Central Midwives Board might be held four, instead of three times a year. There was some discussion on this, Miss Paget pointing out that the Board, which had originally decided to hold four, and then changed it to three examinations yearly, had so determined in order that there might be no stereotyping of three months' training for midwives, but that encouragement to lengthen that term might rather be given. She questioned whether the lying-in hospitals trained many midwives for district work; their pupils were chiefly monthly nurses and hospital nurses completing their curriculum. The shorter term enabled the hospitals to turn out more of these in the year, and the lecturers to get in four courses of lectures, but what was wanted was to aim at a longer training and experience. On a division a resolution acceding to the memorialists' request was carried by five votes to one. The date of the first examination was fixed for June 27th, schedules to be in by June 6th, examinations hereafter to be held in January, April, July, and October.

### Various Questions.

It was agreed to reply to a letter from the Town Clerk of Ramsgate, asking whether the Board would consider the appointment of the Poor-law doctor as inspector of midwives a suitable and proper one, that on the whole the Board considered it would be better not to make such an appointment. The Council of the Association of Poor-law Unions wrote suggesting the reduction from 20 to seven of the cases to be personally delivered, as required by the rules, and the recognition as a training institution of any Poor-law Infirmary where the average number of deliveries per annum amounted to 30. It was agreed in reply to refer the Council to the rules.

The names of 3,976 women applying for certificates under Section 2 of the Act were passed, and ordered for entry on the roll. The total number enrolled amounted to 22,207, of which 12,491 were women in *bonâ fide* practice in July 1901.

The report of the standing committee was considered and adopted. The application of Dorcas Ellenor Ariss to be certified was refused, a communication having been received thereon from Dr. Bateman, secretary of the Medical Defence Union. Martha Maria Herbert, Georgina Eliza Price, and Annie Bord, certified midwives against whom *primâ facie* cases of negligence or misconduct had been proved by the Local Supervising Authorities, were cited to appear before the board. No action was taken with regard to a letter from Dr. J. D. Hendry and R. Hatfield complaining of a circular advertising the services of a certificated midwife attached to the "Regions Beyond Missionary Union Nursing Home," Bow. St. Mary's Hospital, Manchester, was approved as a training school, and a number of medical practitioners were approved as examiners.

The board agreed to the report presented by a sub-committee appointed to consider applications from the secretary and clerk for increase of salary, to be forthwith submitted to the Privy Council for approval. A great deal of business was held over from the Standing Committee, and it was therefore decided to hold an adjourned meeting after the next meeting of the committee, on May 19th; and Thursday, May 25th, was fixed for the hearing of the three "penal cases."

### Novelties for Nurses.

(By Our Shopping Correspondent.)

### WHERE TO BUY A BICYCLE.

Amongst those who really enjoy fresh air and exercise the bicycle has not lost its popularity, although the fashionable craze has passed. Its use amongst district nurses is increasing and becoming more popular, and the lower prices now prevailing, and the facilities offered by many reliable firms render it possible for every nurse, so desiring, to possess one. Messrs. E. O'Brien, of Coventry, make a special study of the supply of bicycles to nurses, and as they have machines of every price and grade, purchasers have only to mention their wants, which Messrs. O'Brien are glad to satisfy. They invite nurses to apply for a catalogue.

### AN ATTRACTIVE NURSES' CATALOGUE.

Nurses are invited to write to Messrs. T. W. Thompson, 164 to 166 Tottenham Court Road, for their fully illustrated catalogue. A graceful and neat cloak is shown on the cover, and within are suggestive designs for in and outdoor uniform, besides a pictorial list of accessories to the nurses' costume. The nurse on her travels is offered an assortment of attractive trunks.

### A Book and its Story.

### A NEW NORTH COUNTRY NOVEL.*

In "Langbarrow Hall" Miss Wilson gives an accurate picture of country life in the Fell district of Westmorland. The local scenery, the dialect, and the hardy types of character found there are presented by one who lives among and understands them with an intelligent recognition. The good and bad points of these dwellers in a part of England which to many persons in the South is an unknown country are faithfully delineated. The story itself centres in Langbarrow Hall. The present owner is Sir Alan de Renegil, who, on the death of his father, finds himself hampered by a determination, which he had formed a few years previously, to build a house which should be worthy of the family traditions in place of the more modest one, with its small windows, thick walls, and old pele tower, which had served for some generations as the home of ancestors conspicuous for physical strength rather than subtlety of brain. The country-side folk prophesied that no good would come of it, and felt some justification for their prognostications when Sir Walter's plans were interrupted by the death of his wife, followed shortly by his own.

" Sir Alan, greatly distressed by his father's sufferings and premature death, had, under an immediate impulse, both inwardly and outwardly vowed to carry out his wishes, so the valley still looked on as the work proceeded. Sir Alan had been born a century too late. He was never truly interested in the mansion building enterprise; he was far happier in his stables or walking the country with his gun under his arm, or satisfying himself regarding the points of his innumerable dogs and horses than in considering architectural devices or planning for life on a more ambitious pattern. What did the size of his house matter? His position was such that it required no artificial elevation. Sir Alan was Sir Alan. What did it matter that every cranny in the old place was crowded with relics, armour, pictures, curiosities, with histories long forgotten? Would they look better in a modern mansion, spread out for the broad stare of a curious upstart rabble?" As a specimen of an English country squire Sir Alan is a good representative of his class—for instance, we read: "Sir Alan took his position as a landowner seriously, and farmed with keen interest. He went in for prize stock himself, and improved that of the valley with lavish neighbourliness He knew the capabilities of every cover, and led the hunt over the Great White Scar with his own hounds, and after his own deer. He sat comfortably in the farmers' kitchens, joked with the men, and described at length, and in minute details, shooting achievements of long past years. Book learning was his abhorrence, and bodily skill in men, beauty in women, and breeding in animals were his standards of excellence. He lavished prizes on the youngsters at the Langbarrow sports, but subscribed to the schools with a grunt of disapprobation." Sir Alan has married recently. His wife, a South-country woman, and as such looked upon as "a foreigner" by his tenants, is described in a scene at the house of his foster-mother, a farmer's wife. Mrs. Dodd's family was inseparably connected with the de Renegils, for members of it had been at the Hall Farm for centuries. Throughout the book she reappears, and her sayings and doings are interwoven with every event of importance. Sir Alan presents his wife to Mrs. Dodd. " Dropping her knitting, she lifted herself out of her chair and met Sir Alan with outstretched arms. 'You see, I've been true to my promise--I've fetched her!' She met a cordial smile from a bright, girlish face, and heard a clear voice speaking: 'How do you do, Mrs. Dodd? My husband told me that you would be one of the first friends I should have the pleasure of seeing!'" Mrs. Dodd's conversation was carried on in dialect, and required interpreting from time to time by Sir Alan. Upon reminding her that they did not talk Westmorland in London, Mrs. Dodd replied, "More shame to them, poor folk! as I tell our postman many a score of times (this in reference to certain new postal regulations "made in London"), I never did reckon nowt of London mysel'." Sir Alan's marriage was a perfectly happy one; but he had secret causes of anxiety to which his wife was a stranger. Money had melted away. The abundant capital left by his father, Sir Walter, for the building of the new mansion, which had accumulated during years of simple living, were ample, but it had been spent in other directions. Racing, betting, and horse-breeding expenses were dissipating his resources, until he finds himself face to face with the fact that he must curtail these expenses if anything in the way of income is to be left for his wife and child. Finding that he is unable to meet the demands which are being made from every quarter by his creditors, he engages, at the advice of his brother, a business man, of very different character to his own, in a speculation which fails. It spells ruin to himself and those dear to him. One night he goes out with his daughter for a walk on the fells and falls over the edge of the scar and is picked up lifeless. So far, only one girl had been born. They had called her Joan. The friendship of she and her boy cousin Réné occupy the leading place in the development of the story.

Réné's sister Bridget—described as being a most perfect type of Saxon womanhood, with violet eyes and long up-tilted lashes; complexion of a rose-petal tint, and soft straight hair, as fine as silk—yet without the same nobility of character as Joan, with all her wild ways. From their school-room days she had looked upon Joan as a rival. She was jealous of her influence over her brother, jealous of her ability and popularity, and moves, with her mother, as the disturbing influence which ultimately wrecks the happiness of more than one person in the story. We must not forget the traditional family portent which is heard when disaster threatens the house of de Renegil in the tolling of the bells of Conishead Priory, nor of a lost codicil to the will, which materially alters the position of Sir Alan's widow, and the baby heir, born after his death. Joan, the joy of her parents, and the outdoor companion of her father until his death, is a natural and bold delineation. Her cousin Réné is unlike her in every way, but they have many sympathies in common. Music is his hobby and art hers. From childhood they have been inseparable companions. Joan is a thorough boy in her love of sport, and Réné follows her lead in everything. Gentle, dreamy, and imaginative, where Joan is practical, bold, and fearless. They have always been child-lovers, and have never had any thought of a future in which they were apart. The curse of the de Renegils pursues the fortunes of the family, and brings the book to a well worked up but tragic close. If, as we believe, "Langbarrow Hall" is Miss Wilson's first essay in novel-writing we wish her every success, for it has much promise. She has shown good sense in writing of persons and things familiar to herself, and the book is one which will be of special interest to readers who are at home| in the scenes described, as well as to those who like a thoroughly wholesome, simply written tale of country life.

* Langbarrow Hall. By T. Wilson Wilson. Harpers. 6s.

### Everybody's Opinion.

[Correspondence on all subjects is invited, but we cannot in any way be responsible for the opinions expressed by our correspondents. No communication can be entertained if the name and address of the correspondent are not given as a guarantee of good faith, but not necessarily for publication All correspondents should write on one side of the paper only.

### SILENCE AT MEALS.

"A Trained Poor-law Infirmary Nurse" writes: Will you give my letter your kind consideration, and, if possible, publish it? I belong to an institution in which there are about 60 nurses, and of these about 40 meet at meals. Under the discipline of a new assistant-matron we are strictly forbidden to talk at table. If by chance one unlucky nurse breaks this rule she is snubbed before all the rest. We are on duty from 7.30 A.M. till 7 P.M. every day without a break, and it seems a very hard matter if when friends meet they may not speak to each other. I have always been under the impression that when nurses are not in the wards the institution is their home. I am quite sure that we should not sit at table in our own homes in such painful silence that one might hear a pin drop. I have been in two other institutions where we were rather encouraged to talk at table, of course providing we were not boisterous. I should like to know whether talking at table is a breach of hospital etiquette, and whether an assistant-matron has power to enforce such a (as it appears to me) most ridiculous rule.

### THE USE OF THE VEIL.

"An Ordinary Nurse" writes: With regard to the veil on bonnets, which is disparaged in your issue of last week, I should like to say that it is a distinctive article of dress, and I have adopted it as a finishing touch to the uniform and a distinction between the dress of a nurse-maid and that of a trained nurse.

### THE QUESTION OF UNIFORM.

"Pension Fund Nurse" writes: I do not wish to fill your paper with yet another long letter on the much-vexed subject of uniform. But as a hospital sister of ten years' standing I should much like to see our "badge of office" protected. I have only one word to say, and that is, Why do the already three-year trained nurses not join the Royal British Nurses' Association and wear the medal on their outdoor uniform? This would, at any rate, be a voucher for many nurses.

"R" writes: I think that Nurse Woodburn Heron must have made a point of observing only the few, the very few, slovenly nurses to be seen in London. I received my training there, and have often visited the metropolis at other times, and almost without exception its nurses have appeared to be neatly dressed and well behaved. I do not think that, taken on the whole, one school of nurses is smarter than another. Bart.'s nurses, no doubt, are nice; but so are Guy's, who, so far as I have seen, always look well dressed and behave as gentlewomen. Of the many less known, perhaps, but equally smart, schools of nurses that could be pointed out for Nurse Heron's benefit I will name only one—the City of London Infirmary, Bow Road.

"An Observer" writes: Your correspondent who s gns herself "Nurse Woodburn Heron" has surely not cultivated that most necessary adjunct to a nurse's character—viz. her powers of observation; but, since she is not a trained nurse, and is, seemingly, devoid of logic, we forgive her. Nurse is ignorant of the fact, perhaps, that countless numbers of nurses eschew wearing the uniform because it most frequently adorns the person of nearly every female who is qualified to push a perambulator, or receive a wage varying from £10 to £18 per annum, and to share her meals with the domestics of a household. They call themselves nurses. They are nurses—in the abstract. Since the uniform of nurses and nurse-maids are similar—indeed, there is no distinguishing mark—nurses have decided to let the nursemaids monopolise the uniform until such a time as the garb can be protected from the abuse it at present sustains from all classes and conditions of females. There are, of course, several hospitals which supply out-door uniform for their staff. This makes the wearing of the uniform, in a measure, compulsory, and embraces all the neatly-dressed, uniformed nurses. The remaining uniformed nurses consist of—(a) women who are desirous of obtaining midwifery training only; (b) raw probationers who do not understand the growing objection of nurses to a garb that is so much patronised by nursemaids; (c) a few members of the profession who are encouraged by personal and economic motives, and steel themselves against the attacks of "hyper-critical" members of the community. It is the trained nurse who should complain of the "outsider"; but perhaps it is more in the nature of things that the "outsider" should make an onslaught upon the rightful owners in order to justify her use of the garb.

### Appointments.

[No charge is made for announcements under this head, and we are always glad to receive, and publish, appointments. The information, to insure accuracy, should be sent from the nurses themselves, and we cannot undertake to correct official announcements which may happen to be inaccurate. It is essential that in all cases the school of training should be given.]

Bridge Schools, Witham.—Miss Alice Williamson has been appointed nurse. She was trained for two years at the Home for Invalids, Highbury, by the Meath Workhouse Nursing Association.

Dufftown Isolation Hospital, Banffshire.—Miss Annie MacConachie has been appointed nurse matron. She was trained at the Western Infirmary, Glasgow, and has since been matron at Stephen Cottage Hospital, Dufftown, and matron at the Northern Infirmary, Inverness.

Hertford British Hospital, Paris.—Miss Ida Nash has been appointed sister. She was trained at the Royal United Hospital, Bath. She has since been on the private staff of the hospital and subsequently night superintendent at the hospital. She holds the certificate of the Central Midwives Board.

Homerton Infirmary.—Miss Ella Louisa Warren has been appointed assistant matron. She was trained at Croydon Union Infirmary, where she has since been staff nurse, and assistant nurse in the lying-in ward. She has also been charge nurse at the General Hospital and Seamen's Infirmary, Ramsgate; charge nurse at Stamford, Rutland, and General Infirmary; charge nurse at the Eastern Fever Hospital, London; and she has served for three years in military hospitals in South Africa and England.

### Where to Go.

Morris's Gallery.—An interesting picture is on view at 14 Duke Street, Manchester Square. This is "The Guards at Waterloo—Hougomont, 1815," by Robert Gibbs, R.S.A. Wellington said: "The success of the Battle of Waterloo turned on the closing of the gates of Hougomont." The momentous struggle is depicted forcibly and dramatically by the artist. The public are invited to view the picture without charge.

### Notes and Queries.

**REGULATIONS.**

**The Editor is always willing to answer in this column, without any fee, all reasonable questions, as soon as possible.**

**But the following rules must be carefully observed.**

1. **Every communication must be accompanied by the name and address of the writer.**
2. **The question must always bear upon nursing, directly or indirectly.**

**If an answer is required by letter a fee of half-a-crown must be enclosed with the note containing the inquiry.**

*Books Required.*

(43) I have been asked to give a nursing lecture to girls. Will you tell me the names of some good up-to-date books on hygiene as I should like to introduce the subject stating the reasons for my statements.—*L. M. E.*

"Practical Elementary Hygiene." By Dr. Furneaux. 2s. 6d. post free. Published by the Scientific Press.

*Six Months' Training.*

(44) Will you tell me if there is any hospital where I could obtain a slight knowledge of general nursing by paying a fee, and could I manage this under six months?—*C. M.*

See "The Nursing Profession: How and Where to Train." You will find that many hospitals will receive you as a paying probationer for three months and upwards, charge about £1 1s. a week. The knowledge of general nursing you could acquire in less than six months would indeed be slight, but much would depend upon yourself.

*Children's Hospital in New South Wales.*

(45) I have been asked to help a children's hospital in New South Wales by writing three letters and sending 10 used British stamps. As the name of the hospital is not even mentioned, do you think that the "snowball" is a genuine one?—*F. M. M.*

Probably so, as no money is asked for, but it would be better to ascertain the name of the Institution.

*Registration.*

(46) I have had three years' fever training in a hospital with over 100 beds. If the Nurses' Registration Bill is passed, am I eligible to register?—*42 Years Old.*

Three years' training in a general hospital with certificate, will probably be necessary to become a registered nurse whenever registration itself is compulsory.

*Rome, or South of France.*

(47) Can you kindly tell me where to write to get particulars about going to either Rome or the South of France. I would like to go as nurse for six months or the season.—*G. F. P.*

In Rome there is the Anglo-American Nursing Home, 265 Via Nomentana, and in Nice, the Nursing Institute, Villa Pilatte, Avenue Désambrois.

*Massage.*

(48) I should be glad to know how to make the best use of my massage training? I was certified for massage and electricity at the National Hospital, Queen Square, and have given massage, electricity, etc., at the Derbyshire Royal Infirmary for 12 months. . . . I am 30 years of age, tall, and strong.—*M. R.*

We can only advise you to advertise or to answer advertisements. Your qualifications as a masseuse are good.

*French Lady.*

(49) Will you kindly tell me if a French lady who has had three years' training in a Dutch hospital could be received in any small hospital or convalescent home in England where she could give her services in return for learning the English language?—*W. A.*

She might advertise, or write to a few matrons of hospitals and convalescent homes. See the "Burdett's Hospitals and Charities." But unless she speaks English fairly well already it is obvious that her services could not be much appreciated. If she could afford to become a paying probationer she would stand a much better chance of being received into a good hospital.

**Handbooks for Nurses.**

| | Post Free. |
|---|---|
| "A Handbook for Nurses." (Dr. J. K. Watson.) ... | 5s. 4d. |
| "The Nurses' Dictionary of Medical Terms." ... ... | 2s. 0d. |
| "Massage" (Swedish.) ... ... ... ... ... | 2s. 6d. |
| "Surgical Ward Work and Nursing." ... ... ... | 3s. 10d. |
| "A Complete Handbook of Midwifery." (Watson.) ... | 6s. 4d. |

Of all booksellers or of The Scientific Press, Limited, 28 & 29 Southampton Street, Strand, London, W.C.

### For Reading to the Sick.

SYMPATHY AND FORBEARANCE.

"Pitiful she was
To all who suffered, measuring loss and woe
By the large measure of her own deep heart,
And by the vastness of its treasure. Thus,
Even through joy, she knew the secret pang
Of sorrow: and through riches—poverty,
And loss by gain."

*B. M.*

God has furnished us with constant occasions of bearing one another's burdens. For there is no man living without his failings; no man that is so happy as never to give offence; no man without his load of trouble; no man so sufficient as never to need assistance; none so wise but the advice of others may, at some time or other, be useful for him; and, therefore, we should think ourselves under the strongest engagements to comfort, and relieve, and instruct, and admonish, and bear with one another.—*Thomas à Kempis.*

We may, if we choose, make the worst of one another. Everyone has his weak points; everyone has his faults; we may make the worst of these; we may fix our attention constantly upon these. But we may also make the best of one another. We may forgive, even as we hope to be forgiven. We may put ourselves in the place of others, and ask what we should wish to be done to us, and thought of us, were we in their place. By loving whatever is lovable in those around us, love will flow back from them to us, and life will become a pleasure instead of a pain; and earth will become like heaven; and we shall become not unworthy followers of Him whose name is Love.—*A. P. Stanley.*

We give much blame, and it may be well. Let us give a little more gratitude, and it will be better for the world. For the world wants kindness far more than harshness. It is very sore with many sorrows, many blows, and we know not how much good a tender voice and a soft hand may do. We have so short a time to live, let us feel and give all the gratitude we can. We shall never regret that in the world beyond, where God is grateful to all who have been kind to His children here.—*Stopford Brooke.*

Talk not of wasted affection, affection never was wasted;
If it enrich not the heart of another, its waters, returning
Back to their springs, like the rain, shall fill them full of refreshment;
That which the fountain sends forth returns again to the fountain.
Patience; accomplish thy labour; accomplish thy work of affection!
Sorrow and silence are strong, and patient endurance is Godlike.
Therefore accomplish thy labour of love, till the heart is made Godlike,
Purified, strengthened, perfected, and rendered more worthy of Heaven.

*Longfellow.*

